# Abdominal Lymphangioma and Hemangioma in a Newborn

**DOI:** 10.1155/2019/6879168

**Published:** 2019-09-17

**Authors:** Charikleia D. Demiri, Christos Kaselas, Dimitrios Godosis, Andreas Neofytou, Ioannis Spyridakis

**Affiliations:** 2 Pediatric Surgery Department, Aristotle University of Thessaloniki, Thessaloniki 54640, Greece

## Abstract

Abdominal hemangiolymphangioma (HLA) in neonates is a rare condition that demands surgical intervention after a complete preoperative diagnostic approach. The differential diagnosis and the establishment of a therapeutic algorithm is a challenge, both for the neonatologists and the pediatric surgeons, because there is no consensus in the management of HLAs in infancy according to the literature. We report a rare case of abdominal HLA in a female newborn that was admitted to our pediatric surgery department with a prenatal diagnosis of an abdominal cystic tumor. After a thorough preoperative diagnostic approach, the neonate underwent an explorative laparotomy and lesion excision with simultaneous splenectomy due to the operative findings. The neonate had an uncomplicated postoperative period and is free of recurrence a year after. Only the pathology examination can reveal the HLA diagnosis. When a total surgical excision is evitable, a close follow-up follows an uncomplicated postoperative hospitalization.

## 1. Introduction

Abdominal tumors in neonates are a challenging issue for pediatric surgeons because of the crucial need for appropriate surgical approach. Infantile lymphangioma and hemangioma (hemangiolymphangioma-HLA) is a rare mixed vascular tumor with both endothelial and lymphatic elements, which belongs to the family of mixed vascular malformations [1, 2]. Approximately 40–60% of HLAs appear at birth and 80–90% during the first 2 years of life, and the frequency decreases with age. The risk of developing HLA is greater in premature babies and in live newborns. The incidence is 1 : 12,000 [3]. HLAs are benign tumors and can be detected at many different anatomical areas such as oral, maxillofacial, and axillary cavity, spermatic cord, scrotum, abdomen, chest, vertebral column, and extremities [4–6]. Here, we present a case of a 3-day-old female neonate who was admitted to our pediatric surgery department with a prenatal diagnosis of an abdominal cystic tumor.

## 2. Case

An otherwise healthy female neonate, the first child of a healthy couple, born with caesarian section at 38 + 4 weeks of gestation with a birth weight of 3200 g, was admitted to our department on the 3rd day of life for evaluation and management of a prenatally diagnosed cystic abdominal mass.

**Figure 1:**
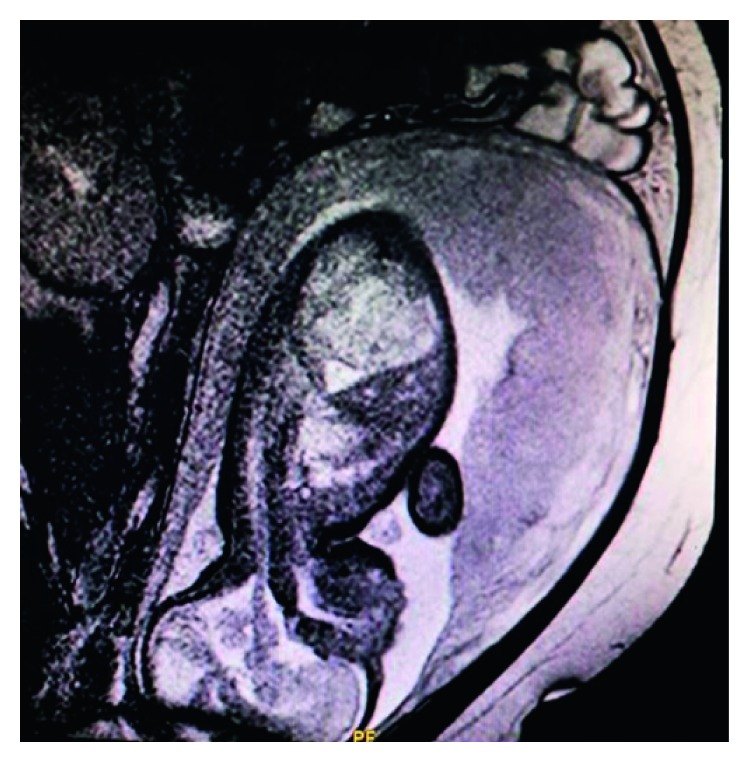
Embryonic MRI that shows the abdominal cystic lesion.

Medical history began at 22 weeks of gestation, when a level-2 ultrasound raised the suspicion of a possible small intestine obstruction. A week later, an embryonic MRI revealed a 25 × 30 × 44 mm cavitary cystic mass with internal hemorrhagic elements. The mass was located in the subdiaphragmatic area, anteriorly but separate from the left kidney, the spleen, and the stomach. Echographic reevaluation at 29 weeks of gestation identified a slight enlargement of the mass (27 × 35 × 59 mm) that persisted during a second embryonic MRI 2 weeks later (see Figure 1).

After birth, a postnatal abdominal ultrasound performed at day 1 of life confirmed the diagnosis and the family was referred to us for further consultation and treatment.

**Figure 2:**
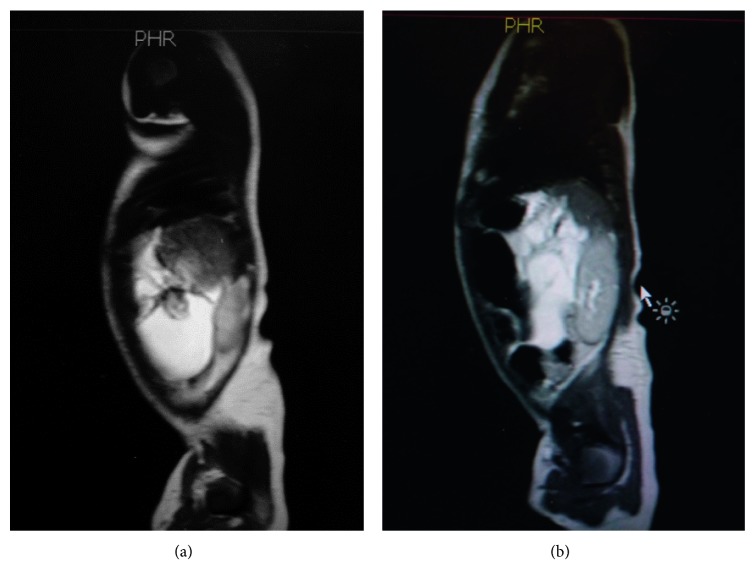
Postnatal MRI depicts the enlargement of the lesion.

At admission, baseline laboratory exams were normal including total *β*-hCG, AFP levels, and urine VMA levels. A new abdominal MRI was performed at day 9 of life and depicted an enlargement of the lesion (37 × 32 × 70 mm), while bone scan and ^123^I-MIBG scan were normal (see Figures 2(a) and 2(b)).

**Figure 3:**
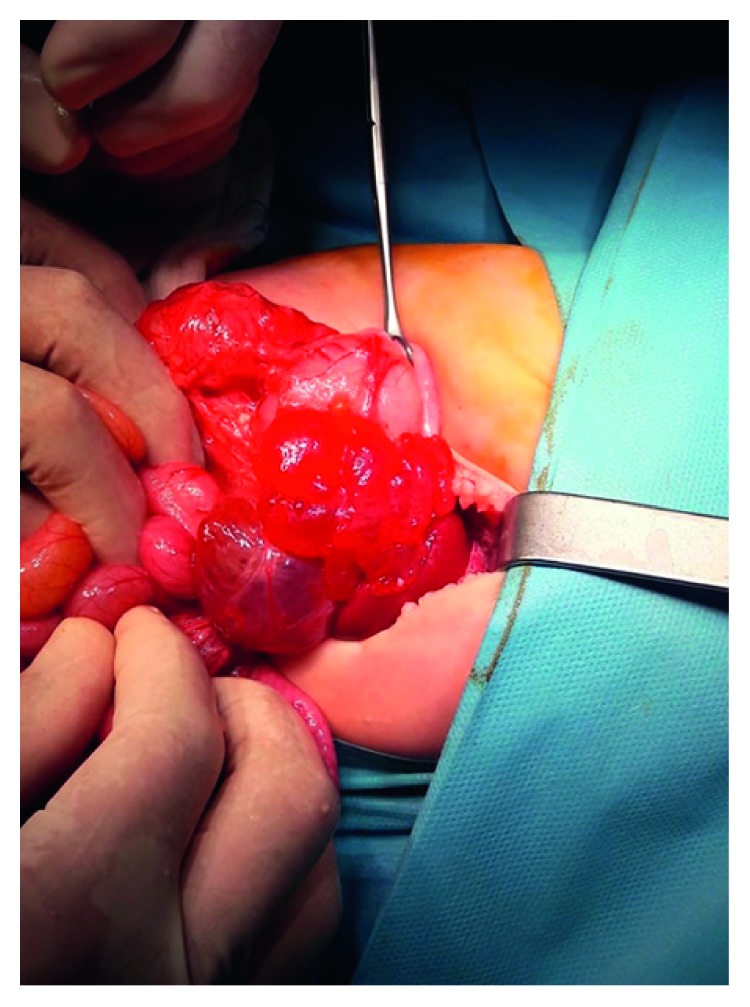
Intraoperative picture that shows the multicystic lesion attached to the spleen.

**Figure 4:**
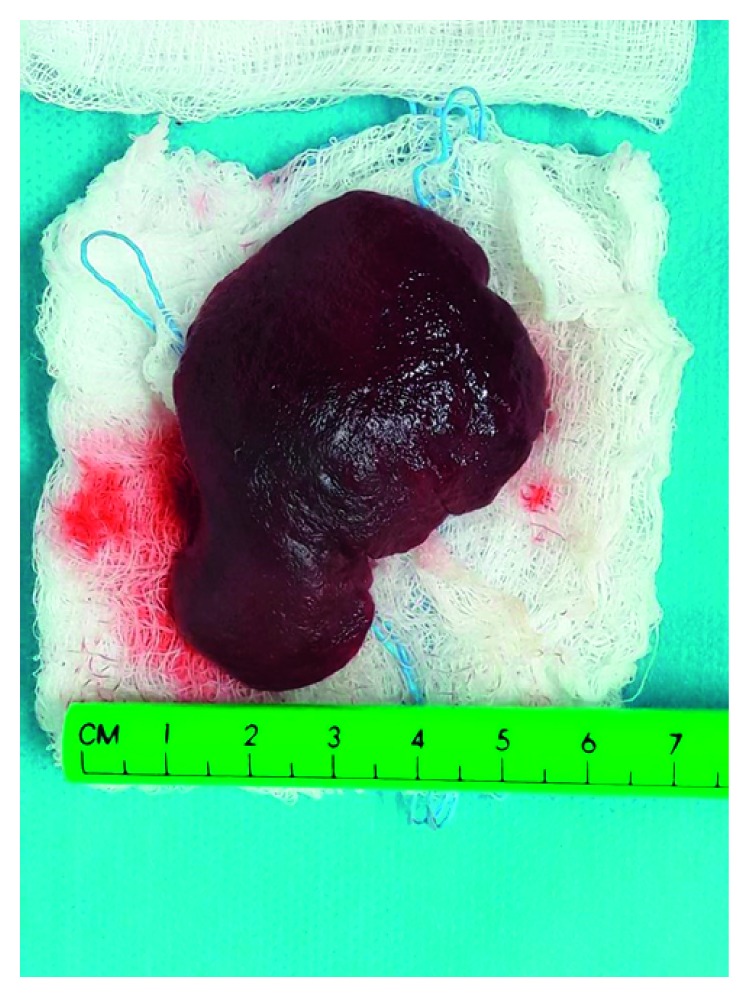
The spleen after the inevitable spleen-sparing operation.

**Figure 5:**
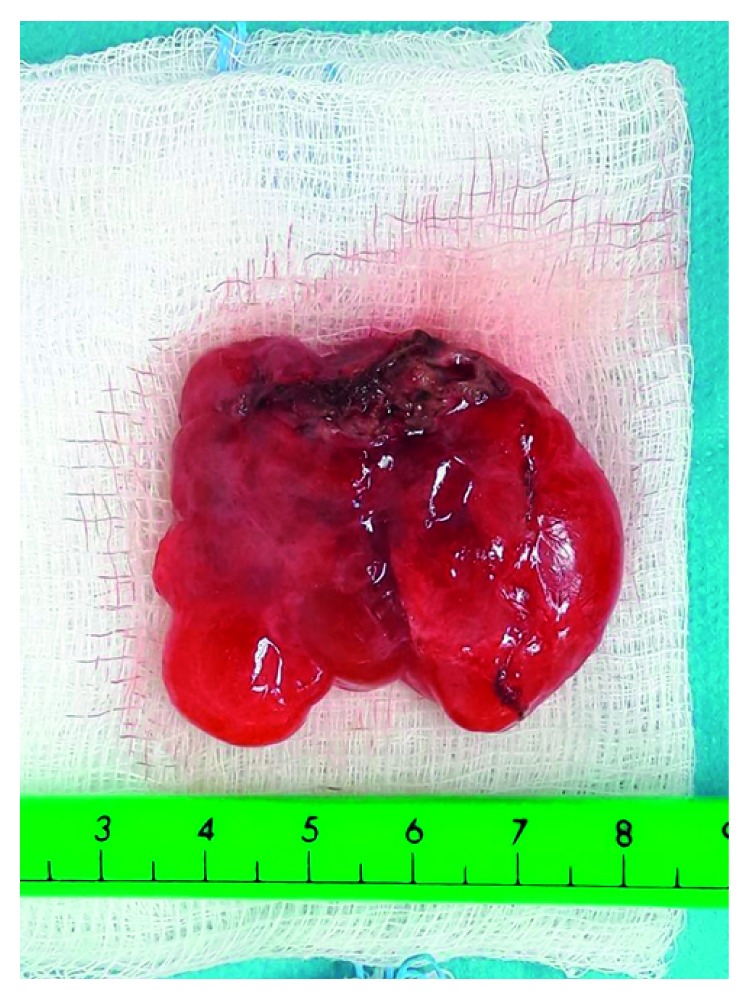
The abdominal LMA.

After the completion of the preoperative investigation, the diagnosis of possible HLA was established and resection of the lesion was decided and performed under general anesthesia. During dissection of the mass, care was taken not to injure the adjacent organs, mainly the spleen and pancreas. However, splenic attachments of the mass were so stiff that a safe complete excision of the mass could not be performed without spleen injury. Therefore, a complete mass excision with splenectomy was decided and performed (see Figures 3–5).

Pathology revealed histopathologic characteristics of neonatal hemangiolymphangioma without mitotic activity or necrosis. The excised spleen was normal.

The neonate had a normal postoperative period and was discharged on the 8th postoperative day under specific chemoprophylaxis and vaccination instructions (due to splenectomy). At the moment, 1 year after the operation, the child remains healthy and asymptomatic. Follow-up included regular abdominal ultrasounds that did not reveal any recurrence.

## 3. Discussion

Abdominal infantile hemangiolymphangioma is an extremely rare condition that calls for a detailed surgical approach because of the risk of a fatal massive bleeding [7].

When concern of an intraabdominal cystic tumor is raised during prenatal ultrasound, an embryonic MRI is proposed as the next step of the diagnostic algorithm. Postnatal abdominal ultrasound and possibly abdominal MRI can confirm the diagnosis.

The differential diagnosis of a newborn with a cavitary abdominal cystic tumor includes cystic lymphangioma, hemangioma, ovarian cyst, cystic teratoma, cystic lesions of the kidney, and cystic neuroblastoma [8, 9]. The heterogeneity of the pathologies mentioned above demands a thorough preoperative clinical examination, laboratory tests, and imaging investigation preoperatively. Safe and complete excision of the lesion is mandatory.

In our case, abdominal HLA was suspected preoperatively and confirmed during histological analysis of the resected specimen that showed both hemangiomatous and lymphangiomatous components, while neither mitotic activity nor necrosis was present.

Etiology and pathogenesis of HLA are not elucidated in the literature. The coexistence of these two pathological entities may be related to abnormal development of the lymphatic structures during the embryonic period [10].

There is no consensus in the management of HLAs in infancy. Main therapeutic approaches include the “wait-and-watch” approach because of the high probability of tumor remission until the age of 18 to 24 months and the surgical approach with complete excision of the lesion. Remission-helping drug components, sclerotherapy, and embolism are described in the literature as alternative pathways [11–15].

When surgical excision is of low risk as in our case, and due to the possibility of mass bleeding, infection, and respiratory distress due to enlargement and space occupation, we prefer the surgical approach in similar cases. We emphasize that each medical case is unique and demands a unique therapeutic approach which should be individualized depending on the size of the lesion, anatomic localization, and possible complications.

## 4. Conclusions

Αbdominal HLA in infancy is an extremely rare condition that demands an adequate surgical intervention because of the high risk of fatal complications. Only the histolopathological examination can reassure the disease. If the prenatal ultrasound raises the concern of an intra-abdominal cystic tumor, an embryonic MRI is proposed as the next step of the diagnostic algorithm. There is no consensus in the management of HLAs in infancy. In our pediatric surgical department, we choose the surgical approach in similar cases as analyzed above. When a total surgical excision is evitable, a close follow-up follows an uncomplicated postoperative hospitalization.
